# E-YOLOv4-tiny: a traffic sign detection algorithm for urban road scenarios

**DOI:** 10.3389/fnbot.2023.1220443

**Published:** 2023-07-18

**Authors:** Yanqiu Xiao, Shiao Yin, Guangzhen Cui, Weili Zhang, Lei Yao, Zhanpeng Fang

**Affiliations:** ^1^College of Mechanical and Electrical Engineering, Zhengzhou University of Light Industry, Zhengzhou, China; ^2^Collaborative Innovation Center of Intelligent Tunnel Boring Machine, Zhengzhou, China

**Keywords:** traffic sign detection, unmanned driving, small object, feature fusion, convolutional neural network, YOLOv4-tiny

## Abstract

**Introduction:**

In urban road scenes, due to the small size of traffic signs and the large amount of surrounding interference information, current methods are difficult to achieve good detection results in the field of unmanned driving.

**Methods:**

To address the aforementioned challenges, this paper proposes an improved E-YOLOv4-tiny based on the YOLOv4-tiny. Firstly, this article constructs an efficient layer aggregation lightweight block with deep separable convolutions to enhance the feature extraction ability of the backbone. Secondly, this paper presents a feature fusion refinement module aimed at fully integrating multi-scale features. Moreover, this module incorporates our proposed efficient coordinate attention for refining interference information during feature transfer. Finally, this article proposes an improved S-RFB to add contextual feature information to the network, further enhancing the accuracy of traffic sign detection.

**Results and discussion:**

The method in this paper is tested on the CCTSDB dataset and the Tsinghua-Tencent 100K dataset. The experimental results show that the proposed method outperforms the original YOLOv4-tiny in traffic sign detection with 3.76% and 7.37% improvement in mAP, respectively, and 21% reduction in the number of parameters. Compared with other advanced methods, the method proposed in this paper achieves a better balance between accuracy, real-time performance, and the number of model parameters, which has better application value.

## 1. Introduction

The semantic information conveyed by traffic signs is essential in providing accurate details on road conditions ahead, which can be used by in-vehicle intelligent systems to help driverless vehicles make informed decisions. Traffic sign detection technology plays a vital role in reducing the incidence of traffic accidents and ensuring safe driving. As a result, it has become a key component of current vehicle-assisted driving systems and holds significant research significance within the urban transportation field (Badue et al., 2021).

Traffic sign detection techniques are divided into traditional methods and deep learning-based methods (Sharma and Mir, 2020). Traditional methods mainly use manually designed features to extract and identify targets by combining multiple features. However, the weak generalization ability of traditional methods for recognition leads to their poor robustness for detection in complex scenes. The algorithms based on deep learning include two-stage detection methods represented by R-CNN (Girshick et al., 2014), Fast R-CNN (Girshick, 2015), and Faster R-CNN (Ren et al., 2015). This type of method first sub-classifies the extracted region candidate frames, and then performs position correction, which results in good detection accuracy. However, the two-stage detection method sacrifices detection speed to a certain extent and requires significant storage space. The YOLO series (Redmon et al., 2016; Redmon and Farhadi, 2017, 2018; Bochkovskiy et al., 2020) and SSD (Liu et al., 2016) are representatives of single-stage detection methods. Instead of omitting the step of generating candidate regions, they directly regress the feature map to obtain target category and boundary box coordinate information. While this approach boasts faster detection speeds compared to the two-stage detection algorithm, it still falls short in terms of accuracy.

The ability to quickly detect distant traffic signs is critical for autonomous driving decision systems to provide sufficient response time. However, traffic signs in images typically occupy an absolute area of no more than 32 × 32 pixels, rendering the detection task a classic example of small target detection (Lin et al., 2014). Currently, many scholars have improved small target detection algorithms based on deep learning. Yang and Tong (2022) proposed a visual multi-scale attention module based on the YOLOv3 algorithm, which integrated feature maps of different scales with attention weights to eliminate the interference information of traffic sign features. Pei et al. (2023) proposed an LCB-YOLOv5 algorithm to detect small targets in remote sensing images. This method improves the accuracy of small target detection by introducing more receptive field and replacing the EIOU loss function. Prasetyo et al. (2022) improved the diversity of network feature extraction by incorporating a wing convolution layer into the YOLOv4-tiny's backbone network. They also added extra detection heads to enhance the accuracy of the detector for small targets. Wei et al. (2023) proposed an approach to improve the detection ability of small targets by adding a transformer attention mechanism and deformable convolution to the backbone network. They also utilized deformable ROI pooling to process multi-scale semantic information extracted from the network, effectively addressing the problem of multi-scale traffic sign detection. Huang et al. (2022) effectively improved the detection accuracy of SSD algorithm for small targets by fusing the target detection layer and adjacent features, and validated it on their own indoor small target dataset. Wu and Liao (2022) proposed a SSD traffic sign detection algorithm combining the receptive field block (RFB) (Liu and Huang, 2018) and path aggregation network (Liu et al., 2018) to improve the target location and classification accuracy. However, this method was only suitable for detection when there was less interference information around the target. While the above-mentioned methods have succeeded in enhancing the detection accuracy of traffic sign models, they have also led to an increase in model size. Since traffic sign detection tasks are usually deployed on devices with limited storage space, the pursuit of lightweight models is of significant practical value.

The backbone serves as the primary feature extractor in a convolutional neural network (CNN) model, and its performance plays a critical role in determining the strength of the model's feature extraction capability. Currently, classic lightweight backbone networks such as the MobileNet series (Howard et al., 2017, 2019), ShuffleNet series (Ma et al., 2018; Zhang et al., 2018), and GhostNet (Han et al., 2020) are widely used. However, although these networks are known for their fast forward reasoning speeds, their feature extraction ability for small targets is suboptimal. On the other hand, more complex backbone networks like ResNet (He et al., 2016), DenseNet (Huang et al., 2017), and DLA (Yu et al., 2018) have achieved higher detection accuracy but at the expense of increased parameter quantity and computational complexity. As a result, these networks may not meet the real-time requirements in terms of reasoning speed. The multi-scale feature fusion method is also an important approach to address the model's insufficient ability to extract small target features. The feature pyramid network (FPN) (Lin et al., 2017) fuses features of multiple scales through top-down lateral connections to obtain fused features with stronger expression capability, which are more beneficial for small target detection. Additionally, other methods such as PANet, NAS-FPN (Ghiasi et al., 2019), and BiFPN (Tan et al., 2020) explore more diverse information fusion paths and adaptive weighting methods to enhance the expression ability of different scale features, further improving the accuracy of small target detection. Although all these methods improve the performance of small target detection to different degrees, they often fail to take into account the significant amount of redundant information present during feature transfer, which can impede the network's ability to effectively fuse multi-scale features.

In urban road scenes, the background of traffic signs often contains many objects with similar characteristics, which will introduce significant interference information during the feature extraction process of the model. This interference feature information may lead to detector misdetection. In recent years, attention mechanisms have emerged as an effective method for enhancing features in the field of deep learning image processing (Guo et al., 2022). By drawing on the process of extracting external information from human vision, the attention mechanism can identify key feature regions of the target from the image and suppress distracting information to enhance representation. Several attention mechanisms, such as SE (Hu et al., 2018), CBAM (Woo et al., 2018), and coordinate attention (Hou et al., 2021), have been proposed to enhance the ability of feature expression by suppressing interference information and capturing key feature areas. However, these methods have limitations. For instance, SE only considers channel weight and ignores location information, while CBAM focuses on local feature information without capturing long-range dependencies. On the other hand, coordinate attention combines both channel and location information and captures long-range dependencies, but at the cost of increased computation, which reduces the real-time performance of the algorithm. Despite these limitations, attention mechanisms have been shown to improve the detection of small targets in complex backgrounds.

In summary, existing deep learning-based methods primarily aim to improve the detection capability of models through enhancing feature extraction, utilizing multi-scale feature fusion, and incorporating attention mechanism. However, in practical applications, traffic sign detection tasks have strict requirements for accuracy, model size, and real-time performance. Existing methods typically focus only on improving detection accuracy, while neglecting model lightweightness and real-time performance, which makes them difficult to be applied in current practical scenarios. In this paper, we propose an E-YOLOv4-tiny algorithm for urban road traffic sign detection based on the current excellent lightweight YOLOv4-tiny (Wang C. Y. et al., 2021) algorithm, from the perspective of achieving a balance among model accuracy, parameter quantity, and real-time performance, and taking into account the influence of interference information in the feature fusion process on multi-scale feature representation. The proposed method can further improve the detection accuracy while reducing the model parameter size, and ensure real-time performance, thereby better application in practical scenarios. The main contributions of this paper are as follows.

(1) To address the poor feature extraction performance of the YOLOv4-tiny's backbone network, we construct a lightweight E-DSC block to optimize it. Drawing inspiration from ELAN's gradient structure and employing depthwise separable convolutions to reduce the network parameters while maintaining performance, we aim to improve the module with minimal parameter costs.

(2) In order to solve the problem of redundant information interference during FPN feature fusion at different levels, a feature fusion refinement module (FFRM) is proposed in this paper. Our method suppresses redundant and interfering information in the multi-scale feature fusion process by constructing a semantic information refinement module and a texture information refinement module that combine efficient coordinate attention (ECA). Additionally, we utilize residual connections to ensure that the output feature maps integrate high-level semantics and detailed information.

(3) We improve the coordinate attention mechanism to further focus and enhance small object features. We use both global max pooling and global average pooling to compress feature maps along the spatial dimension, allowing for a more accurate reflection of channel responses to small objects. Additionally, we employ group convolution and channel shuffling operations to improve the computational efficiency of the model.

(4) To address the issue of limited receptive fields in the YOLOv4-tiny, we propose an S-RFB module in this paper. We simplify the structure of the original RFB module and reduce the number of convolution operations in each branch. The aim is to integrate contextual information into the network to enhance the ability to detect small objects without introducing an excessive number of parameters.

(5) The proposed method in this paper is trained and evaluated on two benchmark datasets, CSUST chinese traffic sign detection benchmark (CCTSDB) and Tsinghua-Tencent 100K (TT100K). The experimental results demonstrate that our method outperforms several state-of-the-art methods in terms of small target detection performance for urban road traffic signs.

The remainder of this article is structured as follows. Section 2 provides a brief overview of the YOLOv4-tiny algorithm. Section 3 provides a detailed introduction to the E-YOLOv4-tiny algorithm proposed in this paper. Section 4 presents the results of ablation experiments conducted on our proposed algorithm, as well as a performance comparison with other state-of-the-art algorithms on CCTSDB and TT100K datasets. Finally, in Section 5, we summarize our article and discuss our future research directions.

## 2. YOLOv4-tiny algorithm

YOLOv4-tiny is a simplified model based on YOLOv4 and is currently a popular model in lightweight detection networks. The detection process of YOLOv4-tiny is the same as YOLOv4. Firstly, YOLOv4-tiny resizes the input image to a fixed size. Next, the input image is divided into candidate boxes, and *S*×*S* small cells are generated for each image. Within each cell, the model predicts *B* boundary boxes and identifies *D*-type objects. The prediction boundary box contains the category name, the center coordinates (*x*,*y*) of the boundary box, the width and height (*w*,*h*) of the boundary box, and confidence information. Finally, a non-maximum suppression algorithm is applied to remove redundant candidate boxes and obtain the final detection boxes for the model.

**Figure 1:**
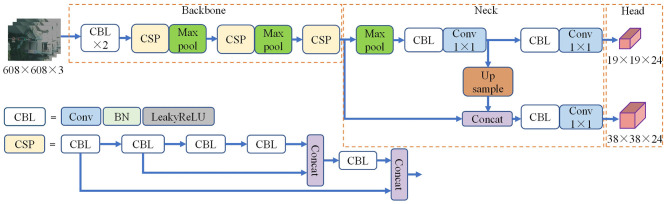
YOLOv4-tiny framework diagram.

The YOLOv4-tiny backbone consists of basic components such as CBL, cross stage partial (CSP) (Wang et al., 2020), etc. The CBL structure consists of a 3 × 3 2D convolution, a BN layer, and a LeakyReLu activation function. In this structure, the convolution kernel step is set to 2 to down-sample the feature map. The CSP structure is made up of the CBL block and the Concat operation. Cross-layer splicing can be used to better connect information about features. YOLOv4-tiny uses the FPN structure for feature fusion and obtains two fusion feature maps with sizes of 38 × 38 and 19 × 19, respectively. Finally, the fusion feature maps are sent to the detection head for processing, and the confidence and position information of the target are obtained. The network structure of YOLOv4-tiny is shown in the Figure 1.

## 3. E-YOLOv4-tiny algorithm

**Figure 2:**
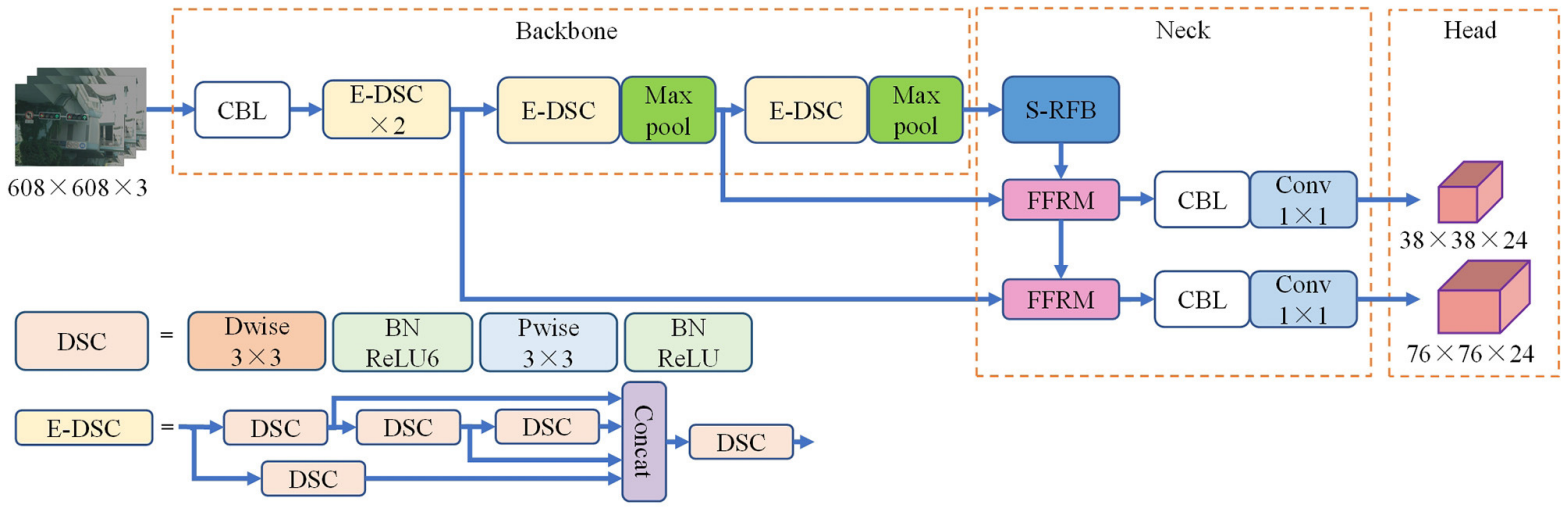
E-YOLOv4-tiny framework diagram.

The YOLOv4-tiny algorithm is not strong in feature extraction for small targets and does not take into account the influence of interference information in feature fusion on multi-scale feature representation, which leads to its low accuracy in detecting traffic signs in urban roads. Therefore, an improved E-YOLOv4-tiny algorithm is proposed in this paper, utilizing feature maps with down-sampling multiples of 4 and 8 as prediction headers to effectively leverage underlying feature maps with more detailed information. Furthermore, the backbone and feature fusion parts are optimized to achieve improved detection performance. Figure 2 illustrates the structure of the E-YOLOv4-tiny.

### 3.1. Backbone based on E-DSC block

The low resolution of traffic signs in images collected from urban roads often presents a challenge for detectors to extract reliable features, which may result in missed targets. ELAN (Wang et al., 2022) addresses the issue of “how to design an efficient network” by analyzing the gradient path of the network. By designing gradient paths in a reasonable manner, ELAN can lengthen the shortest gradient path of the entire network with fewer transition layers, leading to improved efficiency. Furthermore, ELAN combines the weights of different feature layers, enabling the network to learn more diverse features. Compared to CSP structures, ELAN can improve the model's learning capabilities further through better combinations of gradient paths. However, convolution operations in multi-branch paths can significantly increase the network's parameters and consume more memory on the device.

Depthwise separable convolution (DSC) (Howard et al., 2017) can significantly reduce the number of network parameters and computational cost with a small loss of accuracy compared with ordinary convolution, and its structure is shown in Figure 2. DSC partitions the input image into single layer channels and applies depthwise convolution (Dwise) to process spatial information along the long and wide directions. Each channel is associated with a dedicated convolution kernel, and the quantity of channels in the input layer corresponds to the number of feature maps generated. Subsequently, pointwise convolution (Pwise) is applied to supplement the missing cross-channel information in the feature map, leading to the final feature map. Compared with regular convolution, the combination of depthwise convolution and pointwise convolution has the advantage of reducing the number of parameters while ensuring the feature extraction capability.

Based on the aforementioned research, this paper proposes a lightweight E-DSC structure by taking the gradient path in the ELAN structure into account. The goal of this structure is to enhance the network's learning ability without introducing too many parameters, which is achieved through optimizing the stacking of computing modules and fusing deep separable convolutions. The E-DSC structure is illustrated in Figure 2.

This article replaces the CSP with E-DSC in the backbone to enhance the feature extraction ability of the YOLOv4-tiny. The structural details of the improved backbone are shown in Table 1.

**Table 1:** The structural details of the improved backbone.

**Steps**	**Operation**	**Resolution**	**Output channels**	**Number of times**
Input	-	608	3	-
1	CBL	304	32	1
2	E-DSC	152	64	1
3	E-DSC	76	128	1
4	E-DSC	76	256	1
5	Maxpool	38	256	1
6	E-DSC	38	512	1
7	Maxpool	19	512	1
8	S-RFB	19	512	1

### 3.2. Feature fusion refinement module

**Figure 3:**
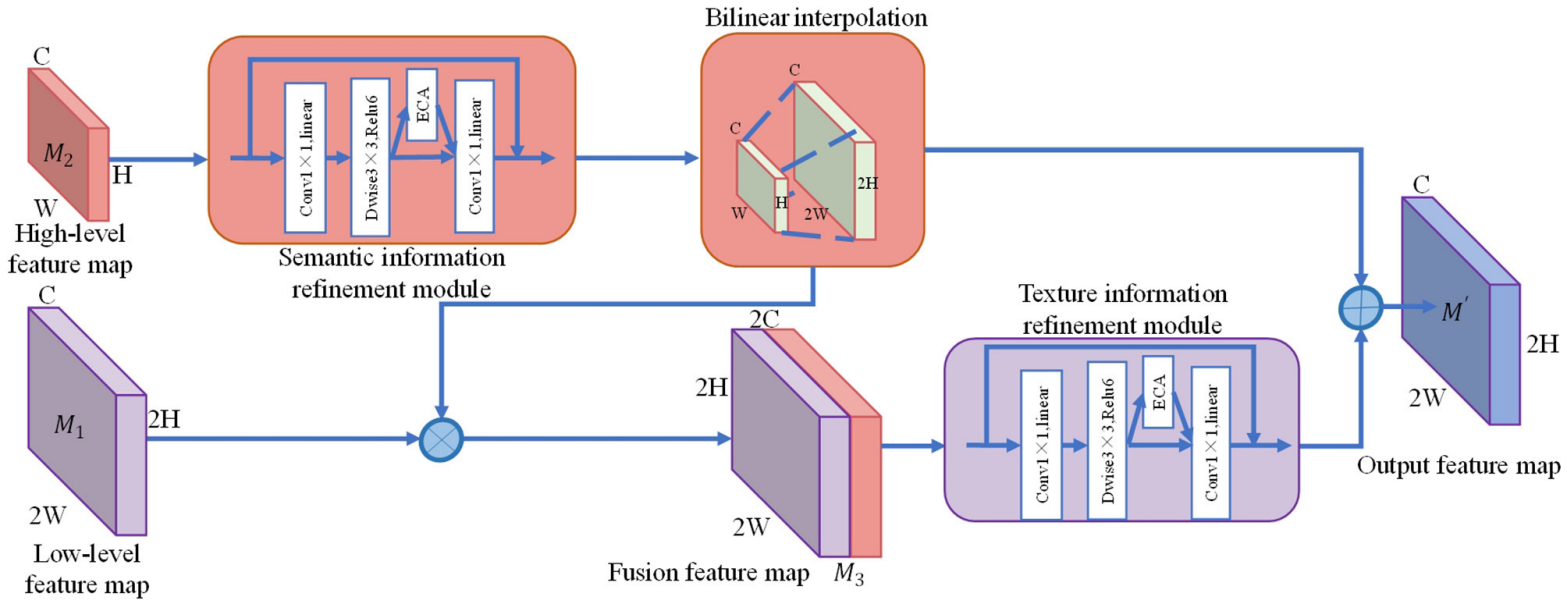
The structure diagram of FFRM.

YOLOv4-tiny uses FPN to fuse feature maps of different scales to predict objects of different sizes, which can improve the overall detection accuracy of the network. In practice, the fusion of feature maps with different scales through up-sampling operations often fails to accurately represent the fused multiscale features due to semantic information differences and interference information. This paper proposes an FFRM to refine and enhance the fused features. The FFRM structure is shown in Figure 3.

In this paper, we leverage the inverted residual structure in Mobilenetv2 (Sandler et al., 2018) and the ECA mechanism proposed herein to construct the semantic information refinement module and the texture information refinement module. These modules are designed to extract semantic and texture information from feature maps of varying scales without introducing too many parameter quantities. This enables the network to learn the significance of feature maps in different channels and spatial dimensions, allowing it to highlight important features while suppressing interference information expression.

The FFRM takes in a low-level feature map ***M***_1_ and a high-level feature map ***M***_2_ as inputs. Firstly, the semantic information refinement module extracts semantic features from ***M***_2_. Secondly, ***M***_1_ is upsampled by using bilinear interpolation and concatenated with ***M***_2_ to obtain the fusion feature map ***M***_3_. Then, the texture information refinement module filters out interference information in ***M***_3_. Finally, an addition operation is used to integrate both high-level semantic information and low-level texture information, resulting in the output feature map ***M***'. The output feature map ***M***' can be represented as:


(1) $$\begin{array}{l} {\text{M}}^{\prime }=R_{T}\left({\text{M}}_{1}⊗\left(R_{C}\left({\text{M}}_{2}\right)\right){↑}_{2\times }\right)⊕R_{C}\left({\text{M}}_{2}\right){↑}_{2\times } \end{array}$$

where *R*_C_ represents the semantic information refine module. *R*_T_ represents a texture information refine module. ⊗ represents concatenate operation. ⊕ represents the element-wise summation operation. ↑_2×_ represents bilinear interpolation up-sampling.

#### 3.2.1. Efficient coordinate attention mechanism

**Figure 4:**
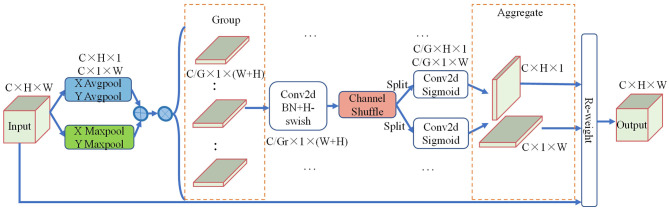
Diagram of efficient coordinate attention.

Traffic signs on urban roads are small in size and are often surrounded by a large amount of background interference information. While the coordinate attention mechanism uses 1D global average pooling to aggregate information from input feature maps, this pooling method only emphasizes the preservation of overall information, which can be challenging to accurately reflect in complex backgrounds for small target information. To address this problem, this paper presents an improved ECA mechanism that utilizes both global average pooling and global maximum pooling to extract the extreme responses of the target channel, allowing for better focus on small target features during down-sampling. This approach enables the network to better capture and highlight the most salient features of the input signal, even in the presence of complex backgrounds and other sources of interference. In addition, embedding the coordinate attention module in the network structure increases the number of parameters, which can reduce detection speed. To address this, this paper introduces the use of group convolution (Krizhevsky et al., 2017) and channel shuffle mechanisms (Zhang et al., 2018) into the structure. These techniques help to further reduce the number of module parameters and computational complexity while maintaining high accuracy. The structure of ECA is shown in Figure 4.

To begin with, each channel of the input feature map **X** ∈ **R**^*C*×*H*×*W*^ are encoded along the horizontal and vertical coordinate directions using the global average pooling and the global maximum pooling with core sizes of (*H*,1) and (1,*W*), respectively. Then, the resulting features in the horizontal and vertical directions are aggregated into four direction-aware feature maps. Thus, the outputs of the *c*-th channel at height *h* can be formulated as:


(2) $$\begin{array}{l} a_{c}^{h}\left(h\right)=Avg\left(\frac{1}{W}\sum_{0\leq i\leq W}^{\sum }x_{c}\left(h,i\right)\right) \end{array}$$

(3) $$\begin{array}{l} m_{c}^{h}\left(h\right)=Max\left(\frac{1}{W}\sum_{0\leq i\leq W}x_{c}\left(h,i\right)\right) \end{array}$$

where *x*_*c*_(*h*,*i*) represents the *c*-th channel component with coordinates (*h*,*i*) in the input feature map ***X***. *Avg* represents the global average pooling. *Max* represents the global maximum pooling. $a_{c}^{h}\left(h\right)$ and $m_{c}^{h}\left(h\right)$ represent the *c*-th channel output at height h after passing through the global average pooling and the global maximum pooling, respectively.

Similarly, the outputs of the *c*-th channel at width *w* can be formulated as:


(4) $$\begin{array}{l} a_{c}^{w}\left(w\right)=Avg\left(\frac{1}{H}\sum_{0\leq j\leq H}x_{c}\left(j,w\right)\right) \end{array}$$

(5) $$\begin{array}{l} m_{c}^{w}\left(w\right)=Max\left(\frac{1}{H}\sum_{0\leq j\leq H}x_{c}\left(j,w\right)\right) \end{array}$$

where *x*_*c*_(*j*,*w*) represents the *c*-th channel component with coordinates (*j*,*w*) in the input feature map ***X***. $a_{c}^{w}\left(w\right)$ and $m_{c}^{w}\left(w\right)$ represent the *c*-th channel output at width *w* after passing through the global average pooling and the global maximum pooling, respectively.

Then, the output components $a_{c}^{h}\left(h\right)$ and $m_{c}^{h}\left(h\right)$, $a_{c}^{w}\left(w\right)$ and $m_{c}^{w}\left(w\right)$ are merged through an element addition operation, as follows:


(6) $$\begin{array}{l} z_{c}^{h}\left(h\right)=a_{c}^{h}\left(h\right)+m_{c}^{h}\left(h\right) \end{array}$$

(7) $$\begin{array}{l} z_{c}^{w}\left(w\right)=a_{c}^{w}\left(w\right)+m_{c}^{w}\left(w\right) \end{array}$$

Then, the two output feature tensors are concatenated in the spatial dimension to generate the feature map **Z** ∈**R**^*C*×1 × (*W*+*H*)^. The feature map **Z** are divided into *G* groups along the channel direction, i.e., **Z** = [**Z**_1_, ..., **Z**_*G*_], ${\text{Z}}_{K}\in {\text{R}}^{C\times 1\times \left(W+H\right)/G}$. The shared 1 × 1 convolutional transformation function *F* is used to reduce the dimension of each group of feature graphs. The process can be formulated as:


(8) $$\begin{array}{l} \text{f}=\delta \left(F\left({\text{Z}}_{K}\right)\right) \end{array}$$

where δ represents the H-swish activation function. **f**∈**R**^*C*×1 × (*W*+*H*)/*G*×*r*^ is the intermediate mapping feature map of group *g*, where *r* is the proportion of the control module size reduction.

Due to the use of group convolution in a continuous manner, boundary effects may occur. That is to say, a small part of the input feature map channel is used for a certain output feature map channel, resulting in no information exchange between different groups and affecting the network's ability to extract global information. Therefore, after obtaining the intermediate feature map, we use the channel shuffle operation to rearrange the order of channels of different group feature maps to achieve intergroup information flow in multiple group convolution layers. In addition, we conducted experiments on the CCTSDB dataset to compare the performance of models with/without channel shuffle, as shown in Table 2.

**Table 2:** Performance comparison of models with/without channel shuffle on the CCTSDB dataset.

**Models**	**mAP@0.5/%**
Baseline	92.44
FFRM (no shuffle)	93.58
FFRM (shuffle)	94.28

The results in Table 2 demonstrate that using the channel shuffle operation in the FFRM module leads to a 0.7% higher mAP metric compared to not using it. This experimental result effectively demonstrates the necessity of using the channel shuffle operation in group convolution, allowing the network to learn more diverse features.

Then, the intermediate mapping feature map is split into two separate feature tensors, **f**^*h*^∈**R**^*C*×*H*×1/*r*^ and **f**^*w*^∈**R**^*C*×1 × *W*/*r*^, along the spatial dimension. Next, the channel numbers of the two tensors are kept consistent with the channel numbers of the input feature map using two convolutional transformations F_*h*_ and F_*w*_, respectively. The process can be expressed by the following formula:


(9) $$\begin{array}{l} {\text{p}}^{h}=\sigma \left(F_{h}\left({\text{f}}^{h}\right)\right) \end{array}$$

(10) $$\begin{array}{l} {\text{p}}^{w}=\sigma \left(F_{w}\left({\text{f}}^{w}\right)\right) \end{array}$$

where σ is the sigmoid activation function.

Finally, the two output tensors are used as attention features, expanded through the broadcast mechanism, and multiplied by the input feature map **X** to give attention weight to obtain the final output feature map **Y**. The process can be formulated as:


(11) $$\begin{array}{l} y_{c}\left(i,j\right)=x_{c}\left(i,j\right)\times p_{c}^{h}\left(i\right)\times p_{c}^{w}\left(j\right) \end{array}$$

### 3.3. S-RFB module

The YOLOv4-tiny network extracts features by using only fixed-size convolutional kernels, resulting in a single receptive field in each layer of the network and making it difficult to capture multiscale information. To address the difficulty of capturing multiscale information using only fixed-size convolutional kernels in YOLOv4-tiny, this study presents an improved version of the receptive field block called S-RFB. The integration of void convolutions with varying expansion rates in S-RFB enriches the extracted features by incorporating rich contextual information and diverse receptive fields into the network. This leads to an improvement in the detection of small traffic sign targets, as the network becomes better equipped to capture and distinguish fine details.

**Figure 5:**
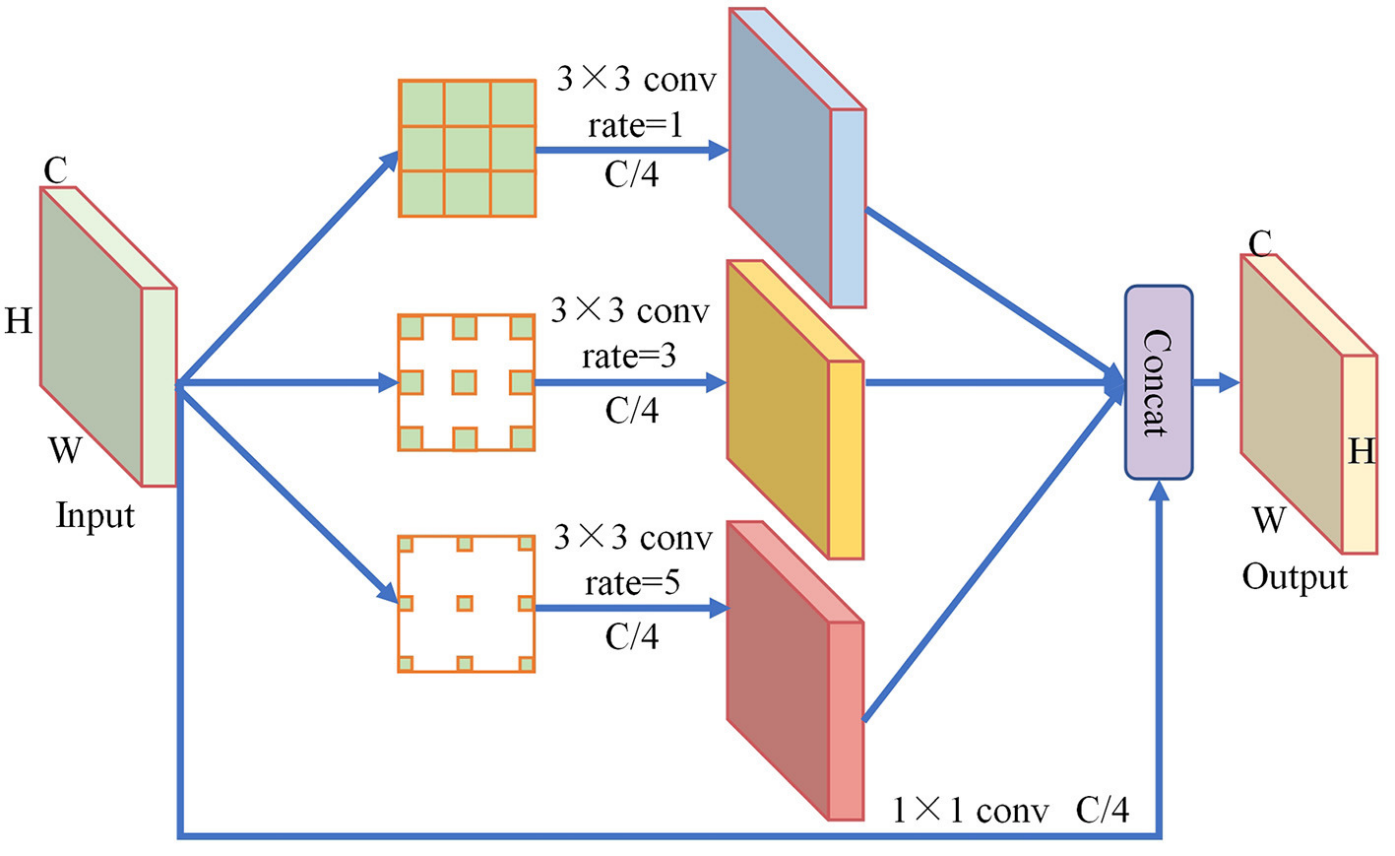
Architecture of S-RFB module.

The structure of the S-RFB module is shown in Figure 5. Firstly, the input feature map with size (*C*,*H*,*W*) is extracted using dilated convolution. The convolution rate is set to 1, 3, and 5, respectively, to obtain three different sizes of receptive fields. To extract more detailed features from the small input feature map of this module, a smaller 3 × 3 convolution is selected in this paper. Meanwhile, the number of convolution kernels is set to *C*/4 to prevent excessive parameters from being introduced. Secondly, a 1 × 1 convolution with a number of *C*/4 is used to concatenate the input feature map, resulting in an equivalent mapping with the output. Finally, the generated feature maps are fused by the Concat operation to aggregate network context information, further enhancing the network's capability to detect small targets.

## 4. Experimental results and analysis

### 4.1. Dataset preparation

This paper initially performs ablation experiments on the CCTSDB dataset (Zhang et al., 2017) to validate the efficacy of each module improvement in enhancing the model's performance. Additionally, the article compares the proposed method with other advanced target detection techniques that currently exist. In addition, this method is also trained and tested on TT100K dataset (Zhu et al., 2016) with richer traffic sign categories and smaller target areas to further verify the generalization ability of this method.

The CCTSDB dataset consists of 13,826 images with nearly 60,000 traffic signs, divided into three categories: mandatory, prohibitory, and warning. Compared to other public traffic sign datasets, this dataset contains mainly urban road scenes with more interference around the targets. This paper divides the dataset into training sets and test sets according to 9:1.

The TT100K dataset consists of images with a resolution of 2,048 × 2048, containing 221 categories of traffic signs with a total of approximately 26,349 targets. Around 40.5% of the total traffic signs have an area of < 32 × 32 pixels, making it crucial for the algorithm to have a high ability to detect small targets. In order to maintain a balanced distribution of target categories, this paper only selects 45 types of traffic signs with more than 100 images for training. A total of 7,968 images are used as the dataset, with 5,289 images used for training and 2,679 images used for testing.

### 4.2. Experimental details

The experimental platform in this article is equipped with an Intel Xeon Sliver 4110 processor with 32GB of memory. There are two NVIDIA Tesla P4 GPUs with 8GB of video memory. The system used in the experiment is Ubuntu 16.04, and the deep learning framework used is Pytorch 1.2.0.

To verify the effectiveness of the algorithm, this paper adopts the same training parameter settings for all network models to ensure experimental fairness. The input image size is set to 608 × 608. The initial learning rate is 0.001. The batch size is 16. The epoch size is set to 500. Select Adam as the optimizer. The cosine annealing algorithm is used in training to attenuate the learning rate.

### 4.3. Evaluation indicators

In the experiment, accuracy (P), recall (R), mean average precision (mAP), frames per s (FPS), and Params are selected to evaluate the performance of the algorithms. The accuracy and recall are used to measure the classification ability and detection ability of the algorithm for targets, and the mAP is used to comprehensively evaluate the detection performance of the algorithm. The formulas for calculating accuracy, recall, and mean average precision are as follows:


(12) $$\begin{array}{l} P=\frac{TP}{TP+FP} \end{array}$$

(13) $$\begin{array}{l} R=\frac{TP}{TP+FN} \end{array}$$

(14) $$\begin{array}{l} mAP=\frac{1}{C}\overset{C}{\underset{i=1}{\sum }}\overset{1}{\underset{0}{\int }}P_{C}\left(R_{C}\right)dR_{C} \end{array}$$

where *TP* indicates that the detection is a positive sample and the result is correct. *FP* indicates that the detection is a positive sample and the result is incorrect. *FN* indicates that the detection is a negative sample and the result is incorrect. *C* represents the number of target categories.

FPS represents the number of frames per second that the network detects images, which is used to evaluate the real-time performance of model. Params refers to the total number of model parameters, and its calculation formula is as follows:


(15) $$\begin{array}{l} params=K_{h}\times K_{w}\times C_{in}\times C_{out} \end{array}$$

where *K*_*h*_ and *K*_*w*_ represent the length and width of the convolution kernel, respectively. *C*_*in*_ and *C*_*out*_ represent the number of convolutional kernel input and output channels, respectively.

### 4.4. Experimental results and analysis

#### 4.4.1. Comparison and analysis of experimental results based on CCTSDB dataset

To assess the effectiveness of the proposed method, we conduct a comparative analysis with five state-of-the-art object detection algorithms on the CCTSDB dataset. Specifically, we evaluate the performance of Faster R-CNN, Centernet (Zhou et al., 2019), SSD, YOLOv5-s (Glenn, 2020), YOLOv4-tiny, and an improved version of YOLOv4 based on attention mechanism (Zhang et al., 2022). Additionally, we add the performance results of YOLOv4-tiny combined with these three different modules to make the part that affects the experimental results more apparent. Specifically, E-DSC, FFRM, and S-RFB represent the models improved using the corresponding methods for YOLOv4-tiny. The evaluation results are tabulated in Table 3.

**Table 3:** Performance comparison results of different models on CCTSDB dataset.

**Models**	**R/%**	**P/%**	**mAP@ 0.5/%**	**Params/ MB**	**FPS/(frame/ s)**
Faster-RCNN	78.84	82.06	84.74	137.09	6
Centernet	65.89	96.94	91.14	32.66	32
SSD	68.15	90.27	76.92	26.28	35
Improved YOLOv4	-	-	96.88	-	40
YOLOv5-s	89.05	92.32	95.11	27.6	42
YOLOv4-tiny	83.81	93.14	92.44	23.1	100
E-DSC	87.05	96.34	94.31	17.6	87
FFRM	88.34	95.08	94.28	25.3	85
S-RFB	84.85	94.49	93.86	23.4	91
E-YOLOv4-tiny	90.14	98.32	96.2	18.2	62

The results reported in Table 3 indicate that the backbone network, as the primary feature extractor of the model, has the greatest impact on the model's performance improvement, with an increase of 1.87% in mAP metric. The algorithm proposed in this paper outperforms advanced two-stage and one-stage algorithms in terms of both accuracy and parameter efficiency. Compared to Faster R-CNN, SSD, Centernet, and YOLOv5-s, the proposed method achieves mAP advantage of 11.46, 19.28, 5.06, and 1.09%, respectively. Moreover, the proposed method improves the mAP index by 3.76% while reducing the number of model's parameters by 21% compared to the original method. The improved YOLOv4 algorithm based on attention mechanism achieves an average detection accuracy of 96.88%, meeting real-time requirements. Additionally, the proposed method in this paper maintains superior detection speed while achieving a similar detection accuracy as the improved YOLOv4 algorithm, effectively demonstrating a good balance between model accuracy and speed.

**Figure 6:**
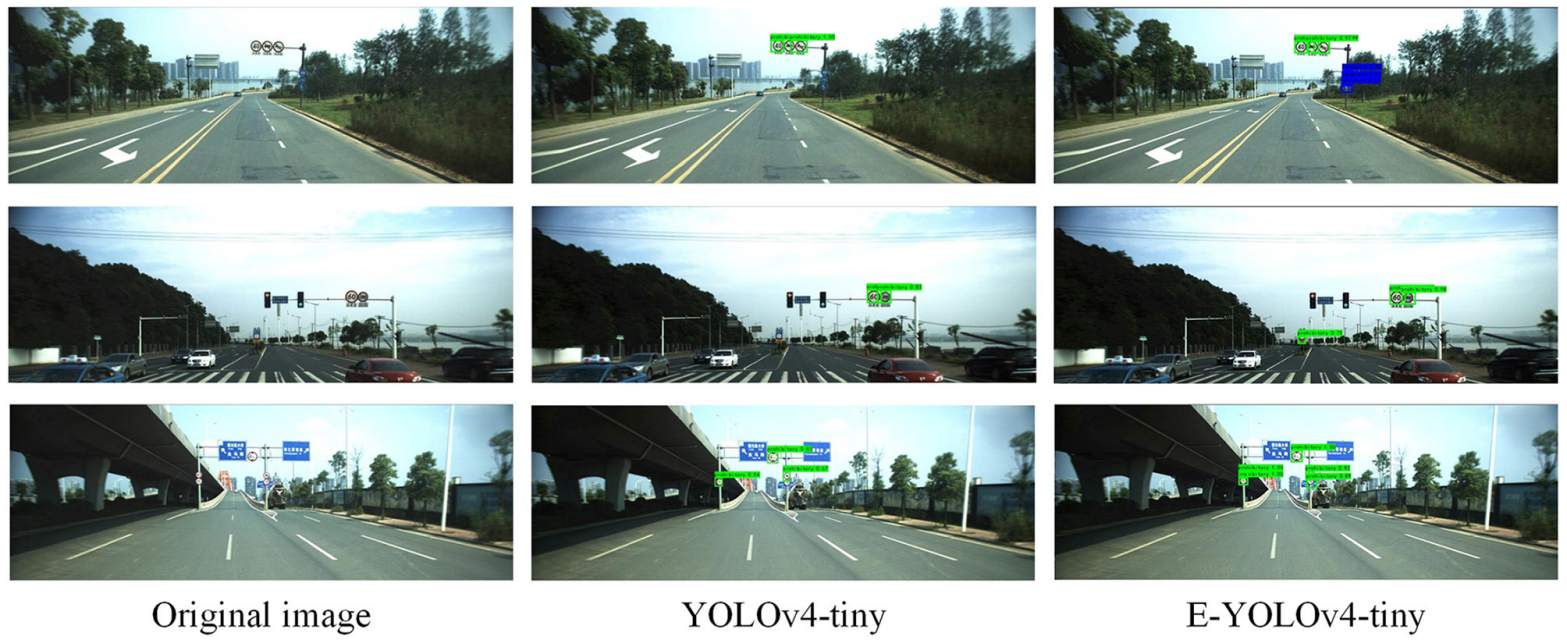
Detection results of CCTSDB dataset.

Figure 6 illustrates the detection performance of our proposed E-YOLOv4-tiny model and the YOLOv4-tiny model on the CCTSDB dataset. The first set of graphs indicates that the E-YOLOv4-tiny model achieves higher confidence levels than the baseline model and can detect three “mandatory” signs that the latter cannot detect. The second set of images demonstrates that our model can still achieve good detection accuracy even in the presence of numerous interfering objects around small targets. In contrast, the YOLOv4-tiny model in the third group of images misses two objects, while our model can detect all objects. These results provide strong evidence that our proposed E-YOLOv4-tiny model outperforms the original YOLOv4-tiny model in detecting small objects.

#### 4.4.2. Comparison and analysis of experimental results based on TT100K dataset

To further validate the generalization ability of our proposed method for detecting traffic signs, we conduct experiments on the TT100K dataset. We compare the performance of our method against several state-of-the-art target detection algorithms, including Fast R-CNN, Centernet, SSD, YOLOv5-s, YOLOv4-tiny, and improved YOLOv4-tiny (Wang L. et al., 2021). At the same time, we add the performance results of YOLOv4-tiny combined with these three different modules. The results of the experiments are presented in Table 4.

**Table 4:** Performance comparison results of different models on TT100K dataset.

**Models**	**mAP@0.5/%**	**Params/ MB**	**FPS/(frame/ s)**
Faster R-CNN	46.23	137.09	6
Centernet	44.09	32.66	32
SSD	40.17	26.28	35
Improved YOLOv4-tiny	52.07	24.7	-
YOLOv5-s	53.28	27.6	42
YOLOv4-tiny	47	23.1	100
E-DSC	50.09	17.6	87
FFRM	49.65	25.3	85
S-RFB	49.27	23.4	91
E-YOLOv4-tiny	54.37	18.2	62

Based on the results presented in Table 4, we observe that the performance of our model on the TT100K dataset is notably lower compared to its performance on the CCTSDB dataset. This difference can be attributed to the smaller absolute area of the targets in the TT100K dataset. In addition, among the compared object detection algorithms, our model achieves the highest mAP value and the lowest Params. Specifically, our proposed method achieves a mAP index of 54.37%, which is 7.37% higher than the original algorithm and 2.3% higher than the improved YOLOv4-tiny. These results demonstrate the effectiveness of our proposed method in this paper.

**Figure 7:**
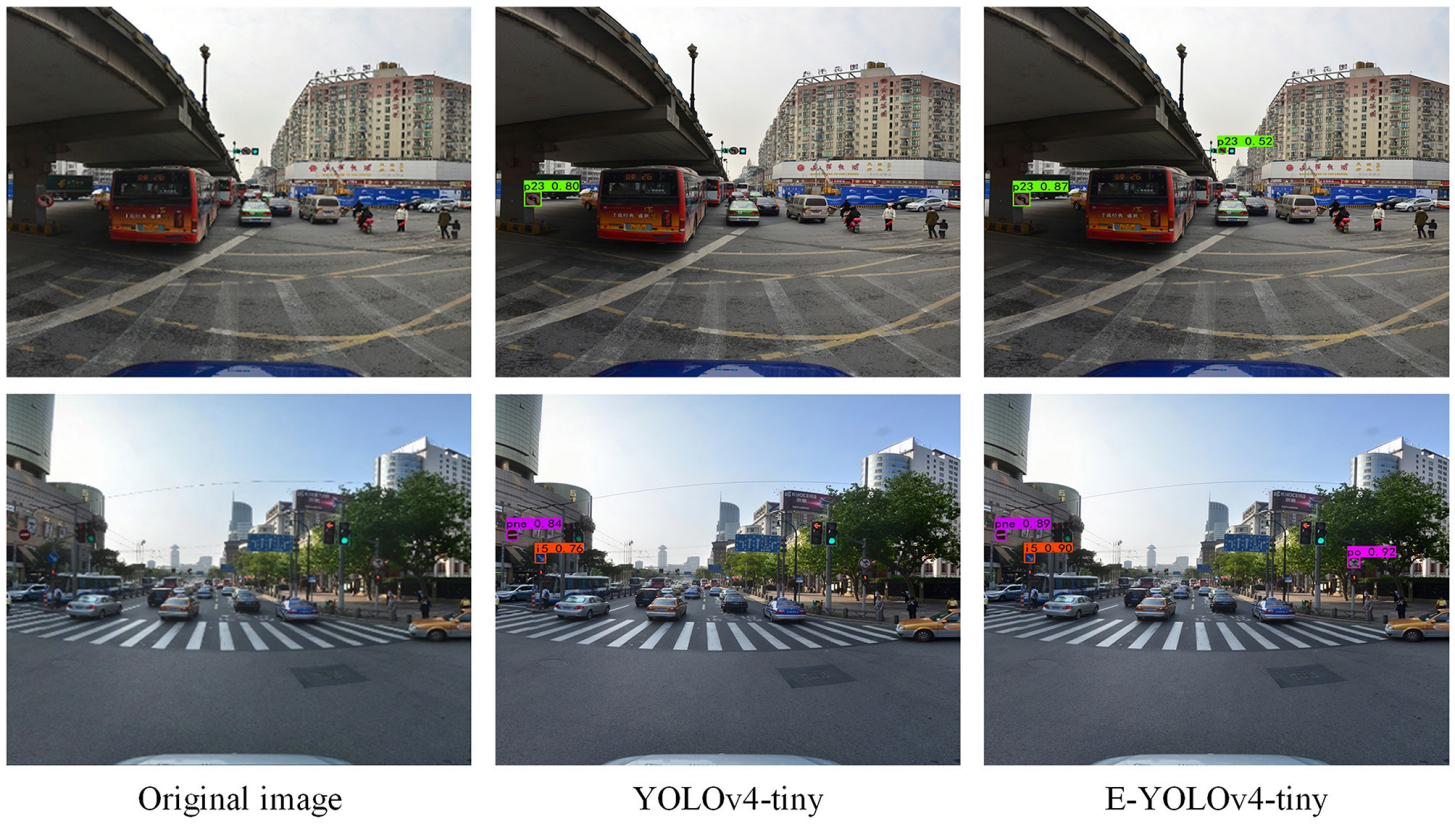
Detection results of TT100K dataset.

In Figure 7, we compare the detection performance of our proposed model with that of the YOLOv4-tiny model on the TT100K dataset. The first set of images demonstrates that our model outperforms YOLOv4-tiny in detecting small traffic signs located at a distance, which indicates that the backbone structure of our model effectively captures the features associated with small targets and reduces the rate of miss detection. In the second set of images, the results indicate that our model can accurately detect three urban road traffic sign targets despite the presence of more interference information around them. Furthermore, our proposed method yields higher confidence scores compared to the original model, providing evidence of its superiority.

### 4.5. Ablation experiments

To assess the effectiveness of our proposed improved method, we conducted ablation experiments on the CCTSDB dataset. We begin by using the YOLOv4-tiny model as a baseline and then add the aforementioned improved methods to enhance its performance. The modified models are trained and tested on the CCTSDB dataset, and the results are compared and presented in Table 5.

**Table 5:** Results of ablation experiments based on CCTSDB dataset.

**Models**	**mAP@0.5/%**	**Params/MB**	**FPS/(frame/s)**
Baseline	92.44	23.1	100
E-DSC	94.31	17.6	87
FFRM	94.28	25.3	85
S-RFB	93.86	23.4	91
E-YOLOv4-tiny	96.20	18.2	62

Comparing the performance indicators of the baseline model and our proposed improved methods in Table 5, we observe that E-DSC, FFRM, and S-RFB are all effective in enhancing the model's mAP performance, with increases of 1.87, 1.84, and 1.42%, respectively. The E-YOLOv4-tiny model, which integrates all three improved methods, achieves the highest mAP performance, with a 3.76% improvement over the baseline. Remarkably, the E-YOLOv4-tiny model also reduces the number of model parameters by 21%, indicating that it is more efficient and cost-effective for practical applications.

**Figure 8:**
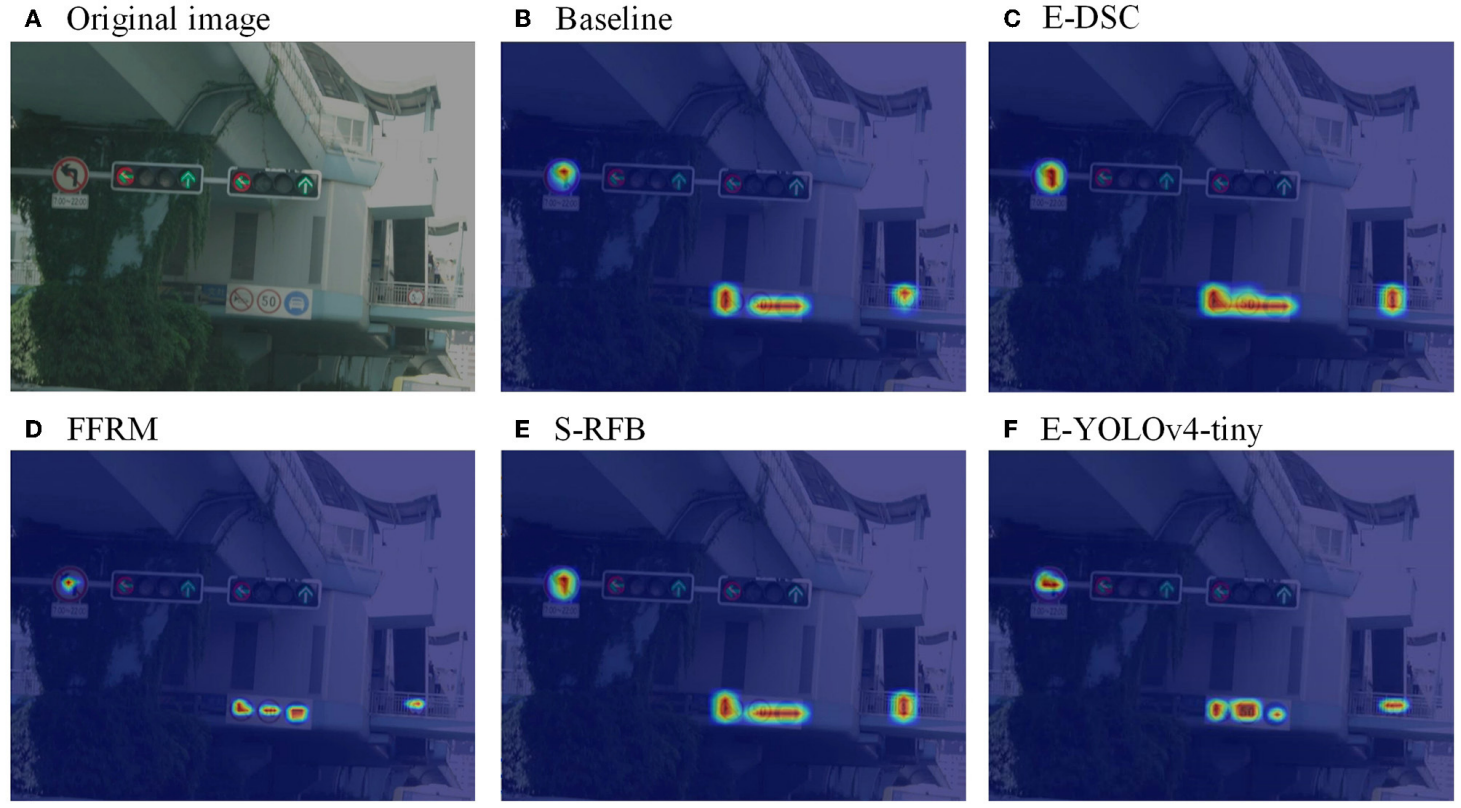
Visualization of heat maps.

We visualize and compare the detection process of each of the above models by heat map for visual comparison, as shown in Figure 8. In the heat map, blue color indicates the minimum activation value for that target region, and red color indicates the maximum activation value for that target region.

The original detection image with four traffic signs, including three “prohibitory” signs and one “mandatory” sign, is shown in Figure 8A. The heat map of the YOLOv4-tiny model is illustrated in Figure 8B, where the activation responses of the “prohibitory” and “mandatory” signs are confused, leading to a higher risk of false detection. Figure 8C shows the heat map of the model based on the E-DSC backbone. The result indicates that the model's ability to extract target features has improved, but there is still confusion in the activation response of similar types of targets. Figure 8D shows the heat map of the model using FFRM in the feature fusion section. It can be seen that the FFRM can enable the network to focus on the main features of the target and effectively distinguish the features of each target. Figure 8E shows the heat map of the model with the addition of the S-RFB context enhancement module, effectively enhancing the model's ability to detect targets. Figure 8F showcases the heat map of the E-YOLOv4-tiny model. The model exhibits distinct activation responses for each target, allowing it to effectively focus on the target feature area and enhance the target area feature activation response.

## 5. Conclusion

In this paper, an E-YOLOv4-tiny traffic sign detection algorithm is proposed to address the difficulties faced by autonomous vehicles in recognizing small target traffic signs in complex urban road environments. Specifically, we address these challenges through three main contributions. Firstly, we propose a lightweight E-DSC block to optimize the backbone and enhance the network's ability to extract small target features. Secondly, we propose an FFRM that fully fuse multi-scale features while efficiently filtering interference information through the ECA. Finally, we introduce an S-RFB module with multi-branch structure and dilated convolutional layer, which can introduce context information into the network and increase the diversity of network's receptive field. The experimental results on the CCTDDB dataset and TT100K dataset demonstrate that our proposed method significantly improves model accuracy and parameter efficiency compared to the YOLOv4-tiny algorithm. Moreover, our method achieves real-time performance, making it highly practical for improving urban road traffic sign detection. Therefore, the advantages of our method lie in achieving a balance between model accuracy, parameter efficiency, and real-time performance, making it more suitable for practical deployment on edge devices for real-time traffic sign detection. Additionally, our proposed method enhances the detection ability of the model for extremely small objects in complex backgrounds present in urban roads. The current drawback of our approach is that, although it achieves real-time performance and outperforms other advanced algorithms, the detection speed still suffers some loss compared to the original algorithm. Furthermore, our method does not consider traffic sign detection in extreme weather conditions. Therefore, our next step will be to research traffic sign detection in extreme scenarios, such as rain, snow, and extreme lighting conditions, in urban roads and minimize the loss of real-time performance as much as possible.

## Data availability statement

Publicly available datasets were analyzed in this study. This data can be found at: CCTSDB: https://github.com/csust7zhangjm/CCTSDB; TT100k: https://cg.cs.tsinghua.edu.cn/traffic-sign/.

## Author contributions

Conceptualization: YX. Methodology: YX and SY. Formal analysis and investigation: GC. Writing–original draft preparation: SY. Writing–review and editing: WZ. Resources: LY. Supervision: ZF. All authors contributed to the article and approved the submitted version.
